# Antibacterial activity in secondary metabolite extracts of heterotrophic bacteria against *Vibrio alginolyticus, Aeromonas hydrophila,* and *Pseudomonas aeruginosa*

**DOI:** 10.12688/f1000research.26215.1

**Published:** 2020-12-21

**Authors:** Jarod Setiaji, Feli Feliatra, Hilwan Yuda Teruna, Iesje Lukistyowati, Indra Suharman, Zainal Abidin Muchlisin, Teuku Iskandar Johan

**Affiliations:** 1 Faculty of Fisheries and Marine Science., Universitas Riau, Pekanbaru, Riau, Indonesia; 2 Faculty of Agriculture, Universitas Islam Riau, Pekanbaru, Riau, Indonesia; 3 Faculty of Mathematics and Natural Science, Universitas Riau, Pekanbaru, Riau, Indonesia; 4 Faculty of Marine and Fisheries, Universitas Syiah Kuala, Banda Aceh, Aceh, 23111, Indonesia

**Keywords:** antibacterial, fish pathogens, heterotrophic bacteria, secondary metabolites

## Abstract

**Background: **Disease causing bacteria such as *Vibrio alginolyticus, Aeromonas hydrophila, *and *Pseudomonas aeruginosa* present a problem for fish farming. Treatment to remove them are generally carried out using antibiotics which have side effects on fish, the environment and humans. However, the use of antibacterial compounds derived from heterotrophic bacteria serve as a good alternative for antibiotics. Therefore, this study aimed to explore antibacterial activity in the secondary metabolite extracts of heterotrophic bacteria against *Vibrio alginolyticus, Aeromonas hydrophila, *and *Pseudomonas aeruginosa*.

**Methods**: Heterotrophic bacteria namely *Bacillus sp.* JS04 MT102913.1, *Bacillus toyonensis* JS08 MT102920.1, *Bacillus cereus* JS10 MT102922.1, *Bacillus *sp. JS11 MT102923.1, *Pseudoalteromonas *sp. JS19 MT102924.1, *Bacillus cereus* JS22 MT102926.1, and *Bacillus *sp. strain JS25 MT102927.1 were used in this study. The sequences of these bacteria have been deposited and are available from NCBI GenBank. Each heterotrophic bacterium was cultured on 6L nutrient broth for 8 days, and extracts produced using ethyl acetate to obtain their secondary metabolites. These extracts were tested for their phytochemical contents using FT-IR and also tested for their inhibitory property in pathogenic bacteria by agar diffusion method.

**Results:** Phytochemical test results showed that the seven heterotrophic bacterial isolates produced terpenoid compounds. Based on the inhibitory test, the secondary metabolite extracts from *Bacillus* sp strain JS04 had the highest inhibitory effect on the growth of pathogenic bacteria namely, *V. alginolyticus *(17.5 mm), *A. hydrophila *(16.8 mm), and *P. aeruginosa* (17.3 mm).

**Conclusion**: It was concluded that the secondary metabolite extracts of heterotrophic bacteria inhibit the growth of *V. alginolyticus, A. hydrophila,* and *P. aeruginosa*.

## Introduction

Bacteria diseases in fish stocks constitute a major problem for fish farming, since they cause significant economic losses ^1– 3^. Common pathogenic bacteria that affect fish include *Vibrio alginolyticus, Aeromonas hydrophila* and *Pseudomonas aeruginosa* ^4– 9^. *V. alginolyticus* is a gram-negative bacteria which is an opportunistic pathogen in marine animals ^10– 13^. Bacterial diseases cause different fish infections such as, exophthalmia, ulcers, septicemia, and corneal damage ^14– 16^. *Aeromonas hydrophila* is found to be the main cause of the septicemia epidemic in freshwater fish ^17, 18^. Its outbreak causes tissue damage of the spleen, gills, and the fish's stomach ^19^. *A. hydrophila* is found to frequently infect various fish species namely, catfish ( *Ictalurus punctatus)* ^20^, carp ( *Cyprinus carpio)* and catfish ( *Pangasius hypophthalmus)* ^21^, tilapia ( *Oreochromis niloticus)* ^22^, salmon ( *Oncorhynchus masou masou)* ^23^, snapper ( *Lates calcarifer)* ^24^, striped snakehead ( *Channa striata)* ^25^, cod ( *Gadus macrocephalus),* and tank goby ( *Glossogobius guris)* ^26^. Meanwhile, *P. aeruginosa* is found to infect freshwater and marine fish ^27, 28^, with infection being characterized by the expression of red spots due to bleeding, skin darkens, loose scales, protruding eyes, fin erosion ^29^, behavioural changes due to disruption of locomotor activity ^30^, and abnormal swimming ^31^.

Bacteria disease treatment is generally carried out using antibiotics, however, these can have adverse effects on the fish and their environment ^32– 37^. The accumulation of antibiotics in the fish increase the risk of bacterial resistance ^38, 39^. *Escherichia coli* bacteria isolated from the digestive organs of catfish showed high resistance levels towards tetracycline, ampicillin, and chloramphenicol ^40^. Therefore, it is necessary to explore natural compounds with antibacterial activity ^41^. Sea water is a potential source of heterotrophic bacteria that produce antimicrobial compound ^42^, and have probiotic activity ^43^

Sea bacteria such as, *Bacillus* sp *. B. cereus, B. toyonensis,* and *Pseudoalteromonas* sp *.*, are known to inhibit the growth of pathogenic bacteria namely, *V. alginolyticus, A. hydrophila,* and *Pseudomonas* sp ^43^. They also produce antimicrobial compounds such as, *Pseudoalteromonas* ^44^. *Pseudoalteromonas piscicida* produces antimicrobial substances that inhibit the growth of different pathogenic bacteria namely, *Vibrio vulniosis* ^45^, *Bacillus* sp ^46^, *B. pumilus* ^47^, and *B. subtilis* ^48, 49^. *Bacillus amyloliquefaciens* shows antibacterial activity towards pathogenic bacteria such as, *Aeromonas hydrophila, Vibrio harveyi, V. vulnificus,* and *V. parahaemolyticus* ^50^. Meanwhile, *Bacillus subtilis* shows antibacterial activity towards the pathogens *Vibrio parahaemolyticus*, *V. vulnificus*, and *Aeromonas hydrophila* ^51^.

Heterotrophic bacteria extracted from Riau sea waters were examined and found to able to inhibit the activity of pathogenic bacteria *Aeromonas salmonicida*, *Edwarsiela tarda* and *Edwarsiela ictaluri* as previously reported by Setiaji *et al*. ^52^. However, the antibacterial activity of these heterotrophic bacteria extracted from Riau sea waters on the pathogenic bacterial namely *, Vibrio alginolyticus, Aeromonas hydrophila,* and *Pseudomonas aeruginos* have never been examined for its potential against pathogenic bacteria. Therefore, this study aims to explore antibacterial activity in secondary metabolite extracts of heterotrophic bacteria isolated from Riau sea water, against pathogenic bacteria namely, *V. alginolyticus, A. hydrophila,* and *P. aeruginosa.*

## Methods

### Bacterial culture

The heterotrophic bacteria isolates were collected from sea waters in Sungai Pakning Bengkalis Regency Riau Province Indonesia (North latitude 01 ^o^21’36,8” and East longitude 102 ^o^09’34,1”). 1 liter of the sea water was collected at 50 cm depth by using a water sampler (Tiolan Lab, type: WSV-BIT22), then was transferred into a sample bottle and was put into a coolbox filled with ice at 15°C, before being transported by car for 1 hour to the laboratory. The heterotrophic bacteria was cultured using nutrient Agar (NA; Merck-1.05450.0500). The heterotrophic bacteria cultured were used for an antagonist test against pathogen bacteria. The antagonist test procedure is as follows, 1 ml of pathogenic inoculants (OD _600nm_ = 0.08–0.1) (OD measured with Thermo scientific, Genesys 10S UV-Vis) was added to 15 ml liquid nutrient Agar media at 50ºC, then homogenized, and poured into a petri dish to solidify. Furthermore, Oxytetracycline antibiotic disc paper (Oxoid, CT0041B, OT30 mcg) was used as the positive control, while 30 µl aquades (Kimiapedia id-1720602804) was dripped to a disc paper (Macherey-nagel, MN827 ATD) as the negative control. 30 µl heterotrophic bacterial isolate taken from bacteria culture in nutrient Broth (NB; Merck-1.05443.0500) was dripped to a disc paper and incubated at 30°C for 24 hours. The inhibitory power of heterotrophic bacterial isolate was measured from the diameter of clear zone formed around the disc. From the antagonist test, eight isolates with the best inhibition were collected, and the heterotrophic bacteria was identified using 16S rDNA technique ^43^. The sequenced products were run through BLAST (NCBI Basic Local Alignment Search Tool) and registered to GenBank.

 The pathogenic bacteria were obtained from the collection at the Marine Microbiology Laboratory of the Faculty of Fisheries and Marine Science, University of Riau, Indonesia. The heterotrophic and pathogenic bacteria were cultured on the nutrient Agar (NA; Merck-1.05450.0500). The cultured medium was sterilized in an autoclave at a pressure of 15 psi and 121°C for 15 minutes. After 1 hour at room temperature, the medium was inoculated by the heterotrophic bacteria and the pathogenic bacteria. Then the bacteria were incubated in an incubator (Memmert, Model 30–1060) at 30°C for 24 hours.

### Isolates test

 Previous studies showed that eight heterotrophic bacterial isolates possessed the potential to produce pathogens. Seven of these species were used in this study namely, *Bacillus sp.* JS04 MT102913.1, *Bacillus toyonensis* JS08 MT102920.1, *Bacillus cereus* JS10 MT102922.1, *Bacillus* sp. JS11 MT102923.1, *Pseudoalteromonas* sp. JS19 MT102924.1, *Bacillus cereus* JS22 MT102926.1 and *Bacillus* sp. strain JS25 MT102927.1 have been deposited in GenBank.

Then, each bacterium was cultured in a 6 L nutrient Broth (NB; Merck-1.05443.0500) diluted with sea water of salinity 29 ppt and aerated for 8 days. After this, the bacteria were mixed with ethyl acetate (P.a) at ratio 1:1 and shaken vigorously to homogenize. Subsequent filtering was performed until a clear filtrate was obtained using funnel and filter paper (Whatman 41, no. 1441–125), and evaporated with a rotary evaporator (Cole Parmer, N-1300) at 50°C and a speed of 50 rpm. This allowed thick secondary metabolite extracts to be obtained ^53^.

### Phytochemical test and Fourier-transform infrared spectroscopy (FT-IR)

Phytochemical test was conducted on the secondary metabolite extracts of heterotrophic bacteria, which included tests for alkaloid, terpenoid, flavonoid, phenolic, and saponin compounds ^54^.

Mayer reagent was prepared by adding 1.36 g HgCl _2_ (Merck, 1.04419. 0050) to 60 mL distillled water and 5 g Ki (Meck 1.05043.1000) to 10 mL distilled water. Both solutions were then mixed with a further 20 mL distilled water. The Liebermen – burchad reagent was prepared by mixing 97% H _2_SO _4_ (Merck 1.00731.2500) and 100% CH _3_COOH (Merck 1.00063.2500).

Alkaloids were tested for using using 10 mg heterotrophic bacteria extract and 250 µL Mayer reagent.

The terpenoid was tested using 10 mg heterotrophic bacteria extract, 10 drops of CH _3_COOH, and 3 drops of H _2_SO _4_.

Flavonoid tests were performed using 10 mg heterotrophic bacteria extract added to 5 mL distilled water. This was then boiled before adding 0.05 g Mg (Merck 1.05815.1000) and 10 drops of 37% HCl (Merck 1.00317. 2500), the mixture was then shaken for one minute.

 Phenolic compounds were tested by using 10 mg heterotrophic bacteria extract combined with 500 µL 5% FeCl _3_ (Merck 1.03943.0250).

Saponin compounds were tested for using 10 mg heterotrophic bacteria extract added to 5 mL distilled water which was then shaken for 1 minute. 150 µL 1N HCl (Merck 1.00317. 2500) was then added, and shaken for another minute.

A positive alkaloid test was indicated by the formation of a white precipitate after adding Mayer regent. A positive terpenoid test was indicated by the formation of a red colour. A positive flavonoid test was indicated by a red colour change. Phenolic compounds were indicated by a blue colour change. Saponin compounds were indicated by a foam forming.

Meanwhile, to determine the functional groups in secondary metabolite extracts, FT-IR (Shimadzu, IR prestige-21, IR solution software ver. 1.1) spectroscopy analysis was performed. This was conducted by crushing 1 mg of each extract, added to KBr (Merck-1.04950.0500), and mixed vigorously until homogenized. This mixture was then measured for infrared absorbance at 4500–450 cm wavelength.

### Inhibitory activity of heterotrophic bacterial extract

The secondary metabolite extracts of heterotrophic bacteria obtained were tested on pathogenic bacteria namely, *V. algynolyticus, A. hydrophila,* and *P. aeruginosa* using agar diffusion method, and 6 mm disc paper (Macherey-nagel, MN827 ATD) ^55^. The procedure is as follows, 1 ml of pathogenic inoculants (OD _600nm_ = 0.08–0.1) (OD measured with Thermo scientific, Genesys 10S UV-Vis) added to 15 ml liquid nutrient agar media at 50°C, then homogenized, and poured into a petri dish to solidify. Furthermore, Oxytetracycline antibiotic disc paper (Oxoid, CT0041B, OT30 mcg) was used as the positive control, while methanol disc paper was the negative control. The metabolite extracts were then dissolved in 1 mg / mL methanol (P.a) and incubated at 30°C for 24 hours. The inhibitory power of heterotrophic bacterial extracts was measured from the diameter of clear zone formed around the disc.

### Data analysis

The data were subjected to one-way analysis of variance followed by the Post Hoc Tukey multiple range test using R 4.0 software (GNU General Public License), *p*<0.05 is considered to indicate a statistically significant difference.

## Results

### Phytochemical test and functional groups

Phytochemical test results of the metabolite extracts when added to Lieberman-Burchard reagents produced a red colour indicating the presence of terpenoids in the seven isolates. Meanwhile, the test for alkaloid, flavonoid, phenolic, and saponin compounds gave negative results.

Based on infrared spectrum analysis, the secondary metabolite extracts of *Bacillus* sp. strain JS04 contained O-H alcohol, C-H aldehyde, O-H carboxylic acid, and C=C alkene groups. *Bacillus toyonensis* JS08 contained C-H alkanes, C=N nitriles, C=O carbonyl, and C-N amine groups. *Bacillus cereus* JS10 contained C-H alkanes, O-H carboxylic acid, C=C alkenes, and C-H alkanes groups. *Bacillus* sp. JS11 contained O-H alcohol, C-H alkanes, O-H carboxylic acids, and C=O carbonyl groups. *Pseudoalteromonas sp.* JS19 contained alcohol O-H, C-H alkanes, C=O carbonyl, and C=C alkenes groups. *Bacillus cereus* JS22 contain O-H alcohol, C-H alkane, C=O carbonyl, and C=C alkene groups. *Bacillus sp*. JS25 contain C-H alkanes, O-H carboxylic acids, O-H alcohols, and C=C alkenes groups ( Table 1 ^56^).

**Table 1.** Infrared spectrum of secondary metabolite extracts of heterotrophic bacteria.

Secondary metabolite extracts	Spectrum (cm ^-1^)	Functional groups
*Bacillus* sp. strain JS04	3148 2732 2535 827	O-H C-H O-H C=C
*Bacillus toyonensis* strain JS08	2925 2361 1722 1229	C-H C=N C=O C-N
*Bacillus cereus* strain JS10	2925 2735 1669 1459	C-H O-H C=C C-H
*Bacillus* sp. strain JS11	3330 2925 2527 1721	O-H C-H O-H C=O
*Pseudoalteromonas* sp. strain JS19	2930 2732 1720 1455	C-H O-H C=O C-H
*Bacillus cereus* strain JS22	3567 2925 1710 827	O-H C-H C=O C=C
*Bacillus* sp. strain JS25	2895 2602 1364 830	C-H O-H O-H C=C

### Inhibitory activity

The results showed that the seven heterotrophic bacterial isolates inhibited the growth of pathogenic bacteria. The extracts inhibitory activity against pathogenic bacteria are shown in Table 2 ^56^. The average inhibition zone diameter of the extracts against pathogenic bacteria namely, *V. alginolyticus, A. hydrophila,* and *P. aeruginosa* ranges from 9.3 to 17.5 mm, 9.3 to 16.8 mm, and 8.5 to 17.3 mm, respectively. This inhibitory zone activity was indicated by the presence of clear zones formed around the disc paper. The largest inhibition zone diameter of the extracts against the growth of pathogenic bacteria was derived from isolates of *Bacillus sp*. strain JS04 (17.5 mm) on *V. alginolyticus*, 17.3 mm on *P. aeruginosa,* and 16.8 mm on *A. hydrophila*.

**Table 2.** Inhibitory activity in the secondary metabolite extracts of heterotrophic bacteria against pathogenic bacteria. Mean values with different superscripts in the same columns were significantly different (p < 0.05).

Secondary metabolite extracts	Average of inhibition zone diameter (mm)		
	*V. alginolyticus*	*A. hydrophila*	*P. aeruginosa*
*Bacillus* sp. strain JS04	17.5 ^a^	16.8 ^a^	17.3 ^a^
*Bacillus toyonensis* strain JS08	11.0 ^b^	9.5 ^b^	10.0 ^b^
*Bacillus cereus* strain JS10	10.8 ^b^	8.5 ^b^	9.5 ^b^
*Bacillus* sp. strain JS11	10.8 ^b^	9.0 ^b^	9.5 ^b^
*Pseudoalteromonas* sp. strain JS19	10.8 ^b^	9.8 ^b^	9.8 ^b^
*Bacillus cereus* strain JS22	9.3 ^b^	9.3 ^b^	8.5 ^b^
*Bacillus* sp. strain JS25	15.8 ^a^	15.5 ^a^	16.3 ^a^

## Discussion

Phytochemical test results showed that the seven heterotrophic bacterial isolates produced terpenoids, which consist 5 carbon atoms or isoprene (C5) units. Microbes carry out biosynthesis by producing isopentyl pyrophosphate and dimethyl allyl pyrophosphate for terpenoid formation ^57^. A significant relationship between terpenoids gene expression and isoprene production in *Bacillus subtilis* has previously been reported ^58^.

Infrared spectrum analysis provided information about the detected compounds in the mixture ^59^. Metabolite extracts showed the presence of hydroxyl, aldehyde, carboxylic acid, alkene, alkane, carbonyl, and amine functional groups in these extracts. This indicated that the seven bacterial isolates produced terpenoids, while the functional groups contained in the terpenoids were namely, O-H hydroxyl, C-H aliphatic, carbonyl, C-H cyclic, and carboxylic acid ^60^.

The result of inhibitory activity in the secondary metabolite extracts of *Bacillus* sp. strain JS04 showed the largest inhibition zone against the growth of pathogenic bacteria. The formation of clear zones on culture media indicated that heterotrophic bacteria produced terpenoid compounds for antibacterial purposes.

The terpenoid compounds contained several phytochemicals that possess antimicrobial activity ^61^. For example, Terpenes and terpenoids have been reported to exert antimicrobial activity against a wide variety of bacteria, both Gram-positive and Gram-negative ^62^. Terpenes cause membrane disruption through acting on lipophilic compound in the membrane ^63^. Therefore, terpenoid compounds were able to prevent the forming of biofilm cell in the bacterium *Streptococcus mutans* ^60, 64^.

 There are many antimicrobial compounds produced by sea bacteria especially from the *Bacillus* and *Pseudoaltreromonas* genus. For instance, *B. pumilus* produces antimicrobial compound against *V. algynolyticus, V. anguillarum, Listeria monocytogenes* and *Staphylococcus aureus* pathogens ^48^ *.* The *Bacillus* sp. from sea water produced chemical compound effective at preventing motility of *V. Algynolyticus* ^47^. *Bacillus subtilis* produced antibacterial compound against *Aeromonas hydrophila* and *Vibrio parahemolyticus* pathogens ^49^. The genus *Pseudoalteromonas* hosts 16 antimicrobial metabolite producers. To date, a total of 69 antimicrobial compounds are classified into alkaloids, polyketides, and peptides ^45^. Furthermore, the bacterium *Pseudoalteromonas rubra* which was symbiotic with soft coral *Sarcophyton* sp *.* produced carotenoid pigments with antibacterial activity against *Staphylococcus aureus* ^65^ and *V. algynolyticus* pathogens ^66^.

## Conclusion

The secondary metabolite extracts produced by the seven isolates of haterotrophic bacteria can inhibit the growth of pathogenic bacteria, namely *V. alginolyticus, A. hydrophila,* and *P. aeruginosa*. The secondary metabolite extracts of *Bacillus sp*. strain JS04 has the highest inhibitory activity against the growth of these three pathogenic bacteria.

## Data availability

### Underlying data

Figshare: Antibacterial activity in secondary metabolite extracts of heterotrophic bacteria against Vibrio alginolyticus, Aeromonas hydrophila, and Pseudomonas aeruginosad Item. https://doi.org/10.6084/m9.figshare.12818798.v3 ^56^

This project contains the following underlying data:

- Data FT-IR activity in the secondary metabolite. Jarod Setiaji.pdf (Infrared spectrum of secondary metabolite extracts of heterotrophic bacteria)
- Data Inhibitory activity in the secondary metabolite. Jarod Setiaji.xlsx (Inhibitory activity in the secondary metabolite extracts of heterotrophic bacteria against pathogenic bacteria)

Data are available under the terms of the Creative Commons Attribution 4.0 International license (CC-BY 4.0).

## References

[1] Cai S Cheng H Pang H: AcfA is an essential regulator for pathogenesis of fish pathogen *Vibrio alginolyticus.* *Vet Microbiol.* 2018;213:35–41. 10.1016/j.vetmic.2017.11.016 29292001

[2] Liu W Huang L Su Y: Contributions of the oligopeptide permeases in multistep of *Vibrio alginolyticus* pathogenesis. *MicrobiologyOpen.* 2017;6(5):e00511. 10.1002/mbo3.511 28714216 PMC5635161

[3] Liu H Gu D Cao X: Characterization of a new quorum sensing regulator luxT and its roles in the extracellular protease production, motility, and virulence in fish pathogen *Vibrio alginolyticus.* *Arch Microbiol.* 2012;194(6):439–452. 10.1007/s00203-011-0774-x 22130678

[4] Chen Y Cai S Jian J: Protection against *Vibrio alginolyticus* in pearl gentian grouper *(Epinephelus fuscoguttatus × Epinephelus lanceolatu)* immunized with acfA-deletion live attenuated vaccine. *Fish Shellfish Immunol.* 2019;86:875–881. 10.1016/j.fsi.2018.12.030 30572128

[5] Zhang D Moreira GS Shoemaker C: Detection and quantification of virulent *Aeromonas hydrophila* in channel catfish tissues following waterborne challenge. *FEMS Microbiol Lett.* 2016;363(9): fnw080. 10.1093/femsle/fnw080 27044300

[6] Stratev D Odeyemi OA: Antimicrobial resistance of *Aeromonas hydrophila* isolated from different food sources: A mini-review. *J Infect Public Health.* 2016;9(5):535–44. 10.1016/j.jiph.2015.10.006 26588876

[7] Lee J Zhang L: The hierarchy quorum sensing network in *Pseudomonas aeruginosa.* *Protein cell.* 2015;6(1):26–41. 10.1007/s13238-014-0100-x 25249263 PMC4286720

[8] Zheng J Tang XM Wu RX: Identification and characteristics of aptamers against inactivated *Vibrio alginolyticus.* *LWT–Food Sci Technol.* 2015;64(2):1138–1142. 10.1016/j.lwt.2015.07.021

[9] Mittal R Khandwaha RK Gupta V: Phenotypic characters of urinary isolate of *Pseudomonas aeruginosa* and their association with mouse renal colonization. *Indian J Med Res.* 2006;123(1):67–72. 16567871

[10] Zhao Z Liu J Deng Y: The *Vibrio alginolyticus* T3SS effectors, Val1686 and Val1680, induce cell rounding, apoptosis and lysis of fish epithelial cells. *Virulence.* 2018;9(1):318–330. 10.1080/21505594.2017.1414134 29252102 PMC5955196

[11] Yu Q Li F Wang Y: Isolation, identification and pathogenicity of *Vibrio alginolyticus* from marine cultured *Trachinotus ovatus.* in Beibu Gulf, Guangxi. *Guangxi Sciences.* 2018;25(1):68–73. Reference Source

[12] Cai SH Lu YS Jian JC: Protection against *Vibrio alginolyticus* in crimson snapper *Lutjanus erythropterus* immunized with a DNA vaccine containing the ompW gene. *Dis Aquat Organ.* 2013;106(1):39–47. 10.3354/dao02617 24062551

[13] Austin B: Vibrios as causal agents of zoonoses. *Vet Microbiol.* 2010;140(3–4):310–7. 10.1016/j.vetmic.2009.03.015 19342185

[14] Wang YD Wang YH Hui CF: Transcriptome analysis of the effect of *Vibrio alginolyticus.* infection on the innate immunity-related TLR5–mediated induction of cytokines in *Epinephelus lanceolatus.* *Fish Shellfish Immunol.* 2016;52:31–43. 10.1016/j.fsi.2016.03.013 26975410

[15] Luo G Huang L Su Y: flrA, flrB and flrC regulate adhesion by controlling the expression of critical virulence genes in *Vibrio alginolyticus.* *Emerg Microbes Infect.* 2016;5(8):e85. 10.1038/emi.2016.82 27485498 PMC5034100

[16] Rameshkumar P Nazar AKA Pradeep MA: Isolation and characterization of pathogenic *Vibrio alginolyticus* from sea cage cultured cobia ( *Rachycentron canadum* (Linnaeus 1766)) in India. *Lett Appl Microbiol.* 2017;65(5):423–430. 10.1111/lam.12800 28901019

[17] Xia H Tang Y Lu F: The effect of *Aeromonas hydrophila* infection on the non specific immunity of blunt snout bream ( *Megalobrama amblycephala*). *Cent Eur J Immunol.* 2017;42(3):239–243. 10.5114/ceji.2017.70965 29204087 PMC5708204

[18] Chaix G Roger F Berthe T: Distinct aeromonas populations in water column and associated with Copepods from estuarine environment (Seine, France). *Front Microbiol.* 2017;8:1259. 10.3389/fmicb.2017.01259 28744262 PMC5504101

[19] Abdelhamed H Ibrahim I Baumgartner W: Characterization of histopathological and ultrastructural changes in Channel Catfish experimentally infected with virulent *Aeromonas hydrophila.* *Front Microbiol.* 2017;8:1519. 10.3389/fmicb.2017.01519 28861049 PMC5559642

[20] Jubirt MM Hanson LA Hanson-Dorr KC: Potential for great egrets (Ardea alba) to transmit a virulent strain of *Aeromonas hydrophila.* among Channel Catfish ( *Ictalurus punctatus*) culture ponds. *J Wildl Dis.* 2015;51(3):634–9. 10.7589/2014-06-156 25984772

[21] Rasmussen-Ivey CR Hossain MJ Odom SE: Classification of a hypervirulent *Aeromonas hydrophila* pathotype responsible for epidemic outbreaks in warm-water fishes. *Front Microbiol.* 2016;7:1615. 10.3389/fmicb.2016.01615 27803692 PMC5067525

[22] Stratev D Stoev S Vashin I: Some varieties of pathological changes in experimental infection of carps ( *Cyprinus carpio*) with *Aeromonas hydrophila.* *Journal of Aquaculture Engineering and Fisheries Research.* 2015;1(4):191–202. 10.3153/JAEFR15019

[23] Han JE Kim JH Choresca C: Draft genome sequence of a clinical isolate, *Aeromonas hydrophila* SNUFPC-A8, from a Moribund Cherry Salmon ( *Oncorhynchus masou masou*). *Genome Announc.* 2013;1(1):e00133–12. 10.1128/genomeA.00133-12 23405367 PMC3569372

[24] Hamid R Ahmad A Usup G: Pathogenicity of *Aeromonas hydrophila* isolated from the Malaysian Sea against coral ( *Turbinariasp*) and seabass ( *Lates calcarifer*). *Environ Sci Pollut Res Int.* 2016;23(17):17269–76. 10.1007/s11356-016-6655-8 27221587

[25] Samayanpaulraj V Sivaramapillai M Palani SN: Identification and characterization of virulent *Aeromonas hydrophila* Ah17 from infected *Channa striata* in river Cauvery and in vitro evaluation of shrimp chitosan. *Food Sci Nutr.* 2020;8(2):1272–1283. 10.1002/fsn3.1416 32148833 PMC7020301

[26] Citterio B Biavasco F: *Aeromonas hydrophila* virulence. *Virulence.* 2015;6(5):417–418. 10.1080/21505594.2015.1058479 26055576 PMC4601520

[27] Mishra P Jerobin SRSJ Thomas J: Study on antimicrobial potential of neem oil nanoemulsion against *Pseudomonas aeruginosa* infection in *Labeo rohita.* *Biotechnol Appl Biochem.* 2014;61(5):611–619. 10.1002/bab.1213 24502533

[28] Thomas J Thanigaivel S Vijayakumar S: Pathogenecity of *Pseudomonas aeruginosa* in *Oreochromis mossambicus* and treatment using lime oil nanoemulsion. *Colloids Surf B Biointerfaces.* 2014;116:372–377. 10.1016/j.colsurfb.2014.01.019 24524941

[29] Altinok I Kayis S Capkin E: *Pseudomonas putida* infection in rainbow trout. *Aquaculture.* 2006;261(3):850–855. 10.1016/j.aquaculture.2006.09.009

[30] Baldissera MD Souza CF Santos RCV: *Pseudomonas aeruginosa* strain PAO1 infection impairs locomotor activity in experimentally infected Rhamdia quelen: Interplay between a stress response and brain neurotransmitters. *Aquaculture.* 2017a;473:74–79. 10.1016/j.aquaculture.2017.02.004

[31] Baldissera MD Souza CF Santos RCV: Blood brain barrier breakdown and myeloperoxidase activity in silver catfish experimentally infected with *Pseudomonas aeruginosa.* *J Fish Dis.* 2018;41(2):209–213. 10.1111/jfd.12697 28836668

[32] Rodriguez-Mozaz S Vaz-Moreira I Giustina SVD: Antibiotic residues in final effluents of European wastewater treatment plants and their impact on the aquatic environment. *Environ Int.* 2020;140:105733. 10.1016/j.envint.2020.105733 32353669

[33] Cao J Zhang J Ma L: Identification of fish source Vibrio alginolyticus and evaluation of its bacterial ghosts vaccine immune effects. *Microbiologyopen.* 2018;7(3):e00576. 10.1002/mbo3.576 29349911 PMC6011932

[34] Chuah LO Effarizah ME Goni AM: Antibiotic Application and Emergence of Multiple Antibiotic Resistance (MAR) in Global Catfish Aquaculture. *Curr Environ Health Rep.* 2016;3(2):118–127. 10.1007/s40572-016-0091-2 27038482

[35] Di Cesare A Luna GM Vignaroli C: Aquaculture can promote the presence and spread of antibiotic-resistant enterococci in marine sediments. *PLoS One.* 2013;8(4):e62838. 10.1371/journal.pone.0062838 23638152 PMC3637307

[36] Romero J Gloria C Navarrete P: Antibiotics in aquaculture-use, abuse and alternatives. *Health and Environment in Aquaculture.* 2012;159–202. 10.5772/28157

[37] Ding C He J: Effect of antibiotics in the environment on microbial populations. *Appl Microbiol Biotechnol.* 2010;87(3):925–41. 10.1007/s00253-010-2649-5 20508933

[38] Rasul MG Majumdar BC: Abuse of antibiotics in aquaculture and it’s effects on human, aquatic animal and environment. *The Saudi Journal of Life Sciences.* 2017;2(3):81–88. 10.21276/haya

[39] Olesen I Hasman H Aarestrup MF: Prevalence of β-Lactamases among ampicillin-resistant *Escherichia coli* and *Salmonella* isolated from food animals in denmark. *Microb Drug Resist.* 2004;10(4):334–340. 10.1089/mdr.2004.10.334 15650379

[40] Damayanti S Kusdarwati R Suprapto H: Bacterial resistance of *Escherichia coli* against antibiotics in *Clarias batrachus* digestion. *AACL Bioflux.* 2019;12(6):2195–2201. Reference Source

[41] Samuel L Marian MM Apun K: Characterization of Escherichia coli isolated from cultured catfish by antibiotic resistance and RAPD analysis. *Int Food Res J.* 2011;18(3):971–976. Reference Source

[42] Alekseeva BI Danilovich KA Valirievna KU: Antimicrobial Activity of Heterotrophic Bacterial Strains of Marine Origin. *Undishapur Journal of Microbiology.* 2013;6(2):166–175. 10.5812/jjm.5039

[43] Setiaji J: DNA Analysis of marine heterotrophic bacteria that are antagonistic towards pathogenic bacteria in fish. *Proceedings of the 18 ^th^ National Ocean Seminar.*Implementation of marine and coastal resources research results in the framework of achieving national economic independence. Faculty of Engineering and Marine Sciences, Universitas Hang Tuah, Surabaya, Indonesia. 2018; c2.17-27. [In Indonesian].

[44] Offret C Desriac F Le Chevalier P: Spotlight on antimicrobial metabolites from the marine bacteria *Pseudoalteromonas*: chemodiversity and ecological significance. *Mar Drugs .* 2016;14(7):129. 10.3390/md14070129 27399731 PMC4962019

[45] Richards GP Needleman DS Watson MA: Whole-Genome Sequences of Two *Pseudoalteromonas piscicida* Strains, DE1-A and DE2-A, with Strong Antibacterial Activity against *Vibrio vulnificus*. *Microbiol Resour Announc.* 2019;8(1):e01451–18. 10.1128/MRA.01451-18 30637397 PMC6318368

[46] Xiu P Liu R Zhang D: Pumilacidin-Like Lipopeptides Derived from Marine Bacterium *Bacillus* sp. Strain 176 Suppress the Motility of *Vibrio alginolyticus*. *Appl Environ Microbiol.* 2017;83(12):e00450-17. 10.1128/AEM.00450-17 28389538 PMC5452807

[47] Zidour M Chevalier M Belguesmia Y: Isolation and Characterization of Bacteria Colonizing *Acartia tonsa* Copepod Eggs and Displaying Antagonist Effects against *Vibrio anguillarum, Vibrio alginolyticus* and Other Pathogenic Strains. * Front Microbiol.* 2017;8:1919. 10.3389/fmicb.2017.01919 29085344 PMC5649146

[48] Chakraborty K Thilakan B Raola VK: Polyketide family of novel antibacterial 7-O-methyl-5'-hydroxy-3'-heptenoate-macrolactin from seaweed-associated *Bacillus subtilis* MTCC 10403. *J Agric Food Chem.* 2014;62(50):12194–12208. 10.1021/jf504845m 25420039

[49] Li W Tang XX Yan X: A new macrolactin antibiotic from deep sea-derived bacteria *Bacillus subtilis* B5. *Nat Prod Res.* 2016;30(24):2777–2782. 10.1080/14786419.2016.1155576 27071303

[50] Chakraborty K Thilakan B Raola VK: Previously Undescribed Antibacterial Polyketides from Heterotrophic *Bacillus amyloliquefaciens* Associated with Seaweed *Padina gymnospora*. *Appl Biochem Biotechnol.* 2017a;184(2):716–732. 10.1007/s12010-017-2562-9 28842846

[51] Chakraborty K Thilakan B Raola VK: Antimicrobial polyketide furanoterpenoids from seaweed-associated heterotrophic bacterium *Bacillus subtilis* MTCC 10403. *Phytochemistry.* 2017b;142:112–125. 10.1016/j.phytochem.2017.06.019 28704687

[52] Setiaji J Feliatra F Teruna HY: Antimicrobial agents derived from heterotrophic bacteria againts pathogenic bacteria. *IOP Conf Ser Earth Environ Sci.* 2019;348:012029 Reference Source

[53] Sateesh VN Rathod JL: Selective isolation and antimicrobial activity of rare actinomycetes from mangrove sediment of Karwar. *Journal of Ecobiotechnology.* 2011;3(10):48–53. Reference Source

[54] Devi GK Manivannan K Thirumaran G: *In vitro* antioxidant activities of selected seaweeds from Southeast coast of India. *Asian Pac J Trop Med.* 2011;4(3):205–211. 10.1016/S1995-7645(11)60070-9 21771454

[55] Feliatra, Muchlisin ZA Teruna HY: Potential of bacteriocins produced by probiotic bacteria isolated from tiger shrimp and prawns as antibacterial to *Vibrio, Pseudomonas*, and *Aeromonas* species on fish [version 1; peer review: 2 approved, 1 approved with reservations, 1 not approved]. *F1000Res.* 2018;7:415. 10.12688/f1000research.13958.1 30363877 PMC6182674

[56] Muchlisin ZA Setiaji J Feliatra F: Antibacterial activity in secondary metabolite extracts of heterotrophic bacteria against Vibrio alginolyticus, Aeromonas hydrophila, and Pseudomonas aeruginosad Item. *figshare.*Dataset.2020 10.6084/m9.figshare.12818798.v3 PMC7839275 33537126

[57] Moser S Pichler H: Identifying and engineering the ideal microbial terpenoid production host. *Appl Microbiol Biotechnol.* 2019;103(14):5501–5516. 10.1007/s00253-019-09892-y 31129740 PMC6597603

[58] Hess BM Xue J Markillie LM: Coregulation of Terpenoid Pathway Genes and Prediction of Isoprene Production in *Bacillus subtilis* Using Transcriptomics. *PLoS One.* 2013;8(6):e66104. 10.1371/journal.pone.0066104 23840410 PMC3686787

[59] Krishna GM Muthukumaran M Krisnhnamoorthy B: A Critical review on fundamental and pharmaceutical analysis of FT-IR spectroscopy. *Int J Pharm.* 2013;3(2):396–402. Reference Source

[60] Widyawati Yanwirasti Djong DH: Potential of terpenoid isolated fromMyrmecodia pendans AS antibacterial against Streptococcus mutans ATCC 2517. *International Journal of Development Research.* 2016;6(11):10350–10354. Reference Source

[61] Barbieri R Coppo E Marchese A: Phytochemicals for human disease: An update on plant-derived compounds antibacterial activity. *Microbiol Res.* 2017;196:44–68. 10.1016/j.micres.2016.12.003 28164790

[62] Mahizan NA Yang SK Moo CL: Terpene Derivatives as a Potential Agent against Antimicrobial Resistance (AMR) Pathogens. *Molecules.* 2019;24(14):2631. 10.3390/molecules24142631 31330955 PMC6680751

[63] Cowan MM: Plant products as antimicrobial agents. *Clin Microbiol Rev.* 1999;12(4):564–582. 10.1128/CMR.12.4.564 10515903 PMC88925

[64] Gartika M Pramesti HT Kurnia D: A terpenoid isolated from sarang semut ( *Myrmecodia pendans*) bulb and its potential for the inhibition and eradication of Streptococcus mutans biofilm. *BMC Complement Altern Med.* 2018;18(1):151. 10.1186/s12906-018-2213-x 29739390 PMC5941495

[65] Wiguna AS Kusmita L Radjasa OK: Antibacterial activity test of carotenoid pigments from soft coral symbiont isolates from *Sarcophyton* sp. on the growth of *Staphylococcus aureus* ATCC 25923 bacteria. *Indonesian Journal of Pharmaceutical Science and Technology.* 2016;3(3):92–98. [In Indonesian]. 10.15416/ijpst.v3i3.9176

[66] Supardy NA Ibrahim D Nor SRM: Bioactive Compounds of *Pseudoalteromonas* sp. IBRL PD4.8 Inhibit Growth of Fouling Bacteria and Attenuate Biofilms of *Vibrio alginolyticus* FB3. *Pol J Microbiol.* 2019;68(1):21–33. 10.21307/pjm-2019-003 31050250 PMC7256726

