# Supplementary material for: Antibacterial activity in secondary metabolite extracts of heterotrophic bacteria against  Vibrio alginolyticus, Aeromonas hydrophila, and  Pseudomonas aeruginosa
Source: F1000Res. 2020 Dec 21;9:1491. [Version 1] doi: 10.12688/f1000research.26215.1 (PMC7839275; doi:10.12688/f1000research.26215.1)
Supplement: Peer review history

# Peer review: Reviewer response for version 1

**DOI:** 10.5256/f1000research.28932.r76451

**Published:** 2021-01-26

**Authors:** Yuhanis Mhd Bakri

**Affiliations:** 1 Department of Chemistry, Faculty of Science and Mathematics, Universiti Pendidikan Sultan Idris, Tanjong Malim, Malaysia

Overall the authors have presented sound article with sufficient findings. Suggestion: Title could be amended to ethyl acetate extracts instead of secondary metabolite extracts. The word inhibitory power in method of inhibitory activity shall be amended appropriately. Isolate tests subtopic shall be amended to extraction. Functional groups investigation is rather general, although acceptable, a further study is needed to verify in length the chemical constituents of secondary metabolites extracts.

Is the work clearly and accurately presented and does it cite the current literature?

Yes

If applicable, is the statistical analysis and its interpretation appropriate?

I cannot comment. A qualified statistician is required.

Are all the source data underlying the results available to ensure full reproducibility?

Yes

Is the study design appropriate and is the work technically sound?

Yes

Are the conclusions drawn adequately supported by the results?

Yes

Are sufficient details of methods and analysis provided to allow replication by others?

Yes

Reviewer Expertise:

Natural product chemistry

I confirm that I have read this submission and believe that I have an appropriate level of expertise to confirm that it is of an acceptable scientific standard.

# Peer review: Reviewer response for version 1

**DOI:** 10.5256/f1000research.28932.r76449

**Published:** 2021-01-14

**Authors:** Agung Damar Syakti

**Affiliations:** 1 Environmental Sciences, Marine Science and Fisheries Faculty, Raja Ali Haji Maritime University (UMRAH), Tanjung Pinang, Indonesia

Evidence of Antibacterial activity in secondary metabolite extracts of heterotrophic bacteria against *Vibrio alginolyticus, Aeromonas hydrophila,* and *Pseudomonas aeruginosa *could be interesting for many applications in aquatic science.  The finding may be useful for Indonesia’s fish farming activities. The study scientifically detailed enough. However, the author(s) should develop much better the occurrence of the specific spectrum thon FT-IR spectra related to the metabolite extracts. The authors should present the FTIR spectra. Thus, the manuscript could be accepted after such minor revision of the spectra figures addition showing the relative response of the functional groups in regard to the spectrum for target compound validation.

Is the work clearly and accurately presented and does it cite the current literature?

Yes

If applicable, is the statistical analysis and its interpretation appropriate?

Partly

Are all the source data underlying the results available to ensure full reproducibility?

Partly

Is the study design appropriate and is the work technically sound?

Yes

Are the conclusions drawn adequately supported by the results?

Yes

Are sufficient details of methods and analysis provided to allow replication by others?

Partly

Reviewer Expertise:

Analytical Environmental Chemistry

I confirm that I have read this submission and believe that I have an appropriate level of expertise to confirm that it is of an acceptable scientific standard.
